# Development of an antibody-ligand fusion protein scFvCD16A_-_sc4-1BBL in *Komagataella phaffii* with stimulatory activity for Natural Killer cells

**DOI:** 10.1186/s12934-023-02082-6

**Published:** 2023-04-11

**Authors:** Yangyang Li, Siqi Xie, Minhua Chen, Hao Li, Yehai Wang, Yan Fan, Kang An, Yu Wu, Weihua Xiao

**Affiliations:** 1 Department of Oncology of the First Affiliated Hospital, Division of Life Sciences and Medicine, University of Science and Technology of China, Hefei, 230027 Anhui China; 2 Hefei National Laboratory for Physical Sciences at Microscale, The CAS Key Laboratory of Innate Immunity and Chronic Disease, School of Life Sciences, University of Science and Technology of China, Hefei, 230027 Anhui China; 3 Institute of Immunology, University of Science and Technology of China, Hefei, 230027 Anhui China; 4 Engineering Technology Research Center of Biotechnology Drugs Anhui, University of Science and Technology of China, Hefei, 230027 Anhui China

**Keywords:** Natural killer cell, Immunotherapy, CD16A, 4-1BBL, *Pichia pastoris*, Antibody-ligand fusion protein

## Abstract

**Background:**

Natural killer (NK) cell-based immunotherapies have demonstrated substantial potential for the treatment of hematologic malignancies. However, its application is limited due to the difficulty in the production of a large number of NK cells in vitro and the insufficient therapeutic efficacy against solid tumors in vivo. Engineered antibodies or fusion proteins targeting activating receptors and costimulatory molecules of NK cells have been developed to encounter these problems. They are mostly produced in mammalian cells with high cost and long processing times. Yeast systems, such as *Komagataella phaffii*, present a convenient manipulation of microbial systems with the key advantages of improved folding machinery and low cost.

**Results:**

In this study, we designed an antibody fusion protein scFvCD16A-sc4-1BBL, composed of the single chain variant fragment (scFv) of anti-CD16A antibody and the three extracellular domains (ECDs) of human 4-1BBL in a single-chain format (sc) with the GS linker, aiming to boost NK cell proliferation and activation. This protein complex was produced in the *K. phaffii* X33 system and purified by affinity chromatography and size exclusion chromatography. The scFvCD16A-sc4-1BBL complex showed comparable binding abilities to its two targets human CD16A and 4-1BB as its two parental moieties (scFvCD16A and monomer ECD (mn)4-1BBL). scFvCD16A-sc4-1BBL specifically stimulated the expansion of peripheral blood mononuclear cell (PBMC)-derived NK cells in vitro. Furthermore, in the ovarian cancer xenograft mouse model, adoptive NK cell infusion combined with intraperitoneal (i.p) injection of scFvCD16A-sc4-1BBL further reduced the tumor burden and prolonged the survival time of mice.

**Conclusion:**

Our studies demonstrate the feasibility of the expression of the antibody fusion protein scFvCD16A-sc4-1BBL in *K. phaffii* with favourable properties. scFvCD16A-sc4-1BBL stimulates PBMC-derived NK cell expansion in vitro and improves the antitumor activity of adoptively transferred NK cells in a murine model of ovarian cancer and may serve as a synergistic drug for NK immunotherapy in future research and applications.

**Supplementary Information:**

The online version contains supplementary material available at 10.1186/s12934-023-02082-6.

## Introduction

Advances in immunotherapy have revolutionized the therapeutic strategy for cancers in the past decade. T cell-based immunotherapy has been at the forefront of cancer immunotherapy, and has been successfully applied to treat a variety of tumors. However, severe side effects limit its application [1–3]. NK cell-based immunotherapy emerged as a safe and effective treatment option for advanced-stage leukaemia as early as twenty years ago, and subsequently made substantial developments. It offers an alternative but much safer option for cancer patients [4–6]. NK cells are the main effector cells of the innate immune system, and are endowed with cytolytic activity and the ability to secrete cytokines and chemokines; thus, they constitute the first line of defense against tumors and pathogens [7]. Owing to their tightly regulated-activated and inhibitory receptors on the membrane, NK cells can kill tumor cells and virus-infected cells while sparing normal cells, without the need for prior sensitization and without major histocompatibility complex (MHC) restriction [8, 9].

Multiple preclinical and early clinical trials, particularly those on hematological malignancy have shown that the adoptive transfer of NK cells is a safe and efficacious treatment [10–13]. Despite the observed progress, NK cell-based therapy still encounters two major challenges: the optimization of clinical-grade, large-scale NK cell expansion with conserved activity in vitro and the improvement of the compromised therapeutic effect on solid tumors [4, 14–17]. At present, pharmacological and genetic strategies have been developed to enhance the clinical therapeutic effect of NK adoptive transfer therapy from different perspectives, such as optimizing the source and expansion approaches of NK cells and enhancing the function and activity of NK cells through gene modification or sstimulatory molecules, i.e. engineering antibodies, NK engagers and antibody-cytokine fusion proteins [14, 16, 18, 19].

CD16 is a low-affinity Fc receptor for IgG, and is responsible for antibody dependent cellular cytotoxicity (ADCC), it contains two subtypes, CD16A and CD16B [20]. CD16A is an important activation receptor expressed on NK cells, macrophages, and mast cells. When CD16A is activated by its ligand or its antibody, it induces a cascade of activation signaling and releases granzymes, perforins, inflammatory cytokines and chemokines, enhancing the proliferation and antitumor activity of NK cells [21–24]. And many activation-related receptors, including 4-1BB (tumor necrosis factor (TNF) receptor superfamily 9, CD137), CD25, CD69, NKG2D, and IL-21R, are upregulated [21, 25–28]. Immobilized anti-CD16 antibodies have been used for PBMC-derived NK cell expansion and activation ex vivo [29, 30]. Some immunotherapy strategies target tumor antigens as well as CD16A receptors to boost NK cell bioactivity, achieving favourable outcomes [31–33].

4-1BB, belonging to the TNF receptor superfamily, is an inducible costimulatory receptor, that is mainly expressed on activated T cells and NK cells [34]. Its ligand 4-1BBL (CD137 L), the only known natural ligand, is expressed on activated antigen presenting cells (APCs), including dendritic cells (DCs), macrophages and B cells [35]. Upon binding to 4-1BBL or agonistic antibodies, 4-1BB leads to a certain activation signaling on NK cells and enhances the antibody dependent cellular cytotoxicity (ADCC) effect of NK cells [25, 36–38]. A previous study showed that immobilized 4-1BBL can expand PBMC-derived NK cells in vitro and that the expanded NK cells have good bioactivity [39]. Anti-CD137 agonistic antibodies have shown therapeutic efficacy in several preclinical and clinical studies [40–42], but they were reported to cause significant hepatic toxicity [41, 43, 44]. 4-1BBL is the natural ligand of 4-1BB, so it may be more appropriate to employ 4-1BBL to activate 4-1BB signalling for cancer treatment.

Fellermeier et al. designed and expressed an antibody fusion protein composed of a tumour-directed antibody linked with three ECDs of 4-1BBL in a single-chain format in a mammalian cell HEK293 expression system. The costimulatory function of the ECDs of this ligand remained conserved [45]. Considering the difficulty in NK cell expansion in vitro and their compromised activities in vivo*,* we designed an immune-stimulatory molecule composed of an NK cell-directed antibody (scFv of CD16A) linked with three ECDs of 4-1BBL in a single-chain format. This fusion protein was produced in *Komagataella phaffii* (*Pichia pastoris*) and its bioactivity was evaluated in vitro and in vivo*.*

*K. phaffii* is a cost-effective eukaryotic expression platform for protein production. Due to its eukaryotic posttranslational modification and efficient secretory production, it has been used to successfully produce distinctive types of proteins, including enzymes, polymers, antigens, and engineered antibody fragments [46, 47]. Compared with mammalian cell expression platforms, *K. phaffii* is more convenient for genetic modifications and carrying out controllable high-cell-density fermentation at a low cost. These unique advantages endow *K. phaffii* with great biopharmaceutical application value [48–50]. Our study demonstrates for the first time the feasibility of expressing of an antibody-ligand fusion protein in *K. phaffii* with the desired bioactivity in vitro and in vivo. We showed that the scFvCD16A-sc4-1BBL complex can stimulate the activation and proliferation of PBMC-derived NK cells and that expanded NK cells are cytotoxic to K562 tumor cells in vitro. Furthermore, adoptive transfer of NK cells in combination with i.p injection of scFvCD16A-sc4-1BBL further inhibited tumor growth and prolonged the survival time of the mice bearing ovarian cancer tumors, which is comparable to the effect of adoptively transferred NK cells with a high dose of rhIL-2 (50,000 IU/mouse).

## Results

### Molecular design and expression of scFvCD16A-sc4-1BBL

**Fig. 1:**
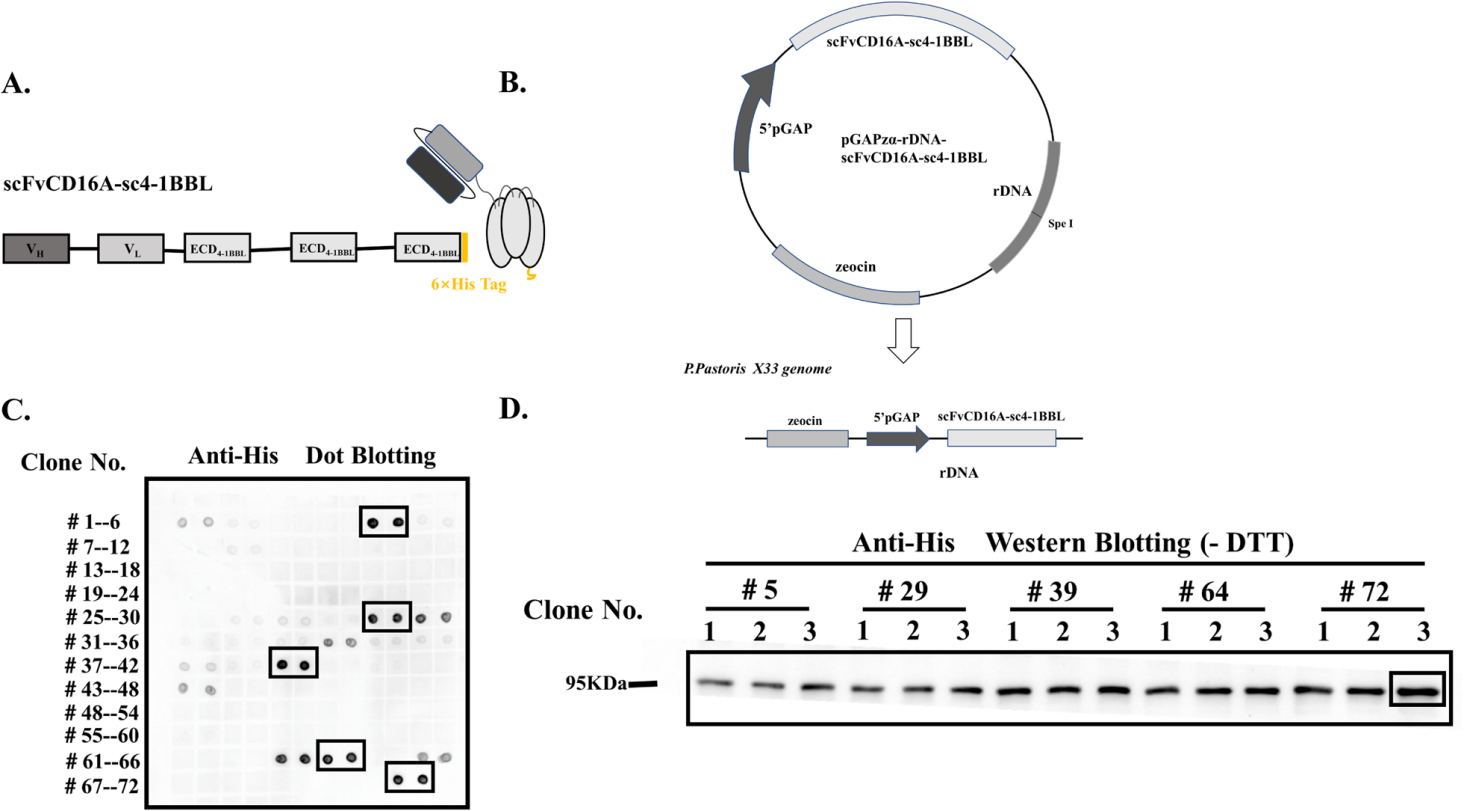
Construction and expression of scFvCD16A-sc4-1BBL.** a** Schematic diagram of the molecular structure scFvCD16A-sc4-1BBL. scFvCD16A-sc4-1BBL was composed of a single chain variable fragment of human CD16A antibody, three extracellular domains (ECDs) of 4-1BBL and a 6X His tag at the C-terminus. **b** The coding sequence of scFvCD16A-sc4-1BBL was inserted into the pGAPzα-rDNA plasmid under the control of the GAP promoter, which then integrated into the genome of *K. phaffii* X33 through homologous recombination. **c** The positive colones expressing scFvCD16A-sc4-1BBL were screened by dot blotting using an anti-His tag HRP conjugate antibody. **d** The high expression clones were further identified by Western blotting under nonreducing conditions (-DTT) using the same antibody as **c**. The clone of No. # 72–3 showed the highest expression and was selected for the following study

The scFvCD16A-sc4-1BBL complex was composed of a scFv of the human CD16A antibody [32], three ECDs of human 4-1BBL and a 6 × His tag at the C-terminal (Fig. 1a). The scFvCD16A antibody was selected to target and activate NK cells. Previous study have shown that the format of the homotrimer ECD of human 4-1BBL has better biological activity than the monomer [45]; thus, we used a homotrimer format connected with glycine-serine (GS) here (Fig. 1a).The two moieties were also connected with length-optimized GS linker [45].

The coding sequence of scFvCD16A-sc4-1BBL was optimized to fit the codon usage bias of *K. phaffii,* synthesized and then inserted into the pGAPzα vector containing a functional bleomycin gene which was labeled as Zeocin in the vector map (Fig. 1b). This vector was inserted into the genome of the X33 strain through homologous recombination. The scFvCD16A-sc4-1BBL gene was fused with the α-factor signal sequence and under the control of the GAP promoter (Fig. 1b).

To obtain a high-expression strain for the scFvCD16A-sc4-1BBL complex, the expression vector pGAPzα-scFvCD16A-sc4-1BBL was transformed into *K. phaffii* strain X33 via electroporation and then spread on a YPD plate containing 300 µg/ml zeocin. The high-expression clones of the scFvCD16A-sc4-1BBL complex were first screened by dot blotting, and then the selected clones were further identified by Western blotting. One of these high-expression clones (#72–3) was selected for the following pilot-scale fermentation (Fig. 1c and d).

### Fermentation, purification and identification of scFvCD16A-sc4-1BBL

**Fig. 2:**
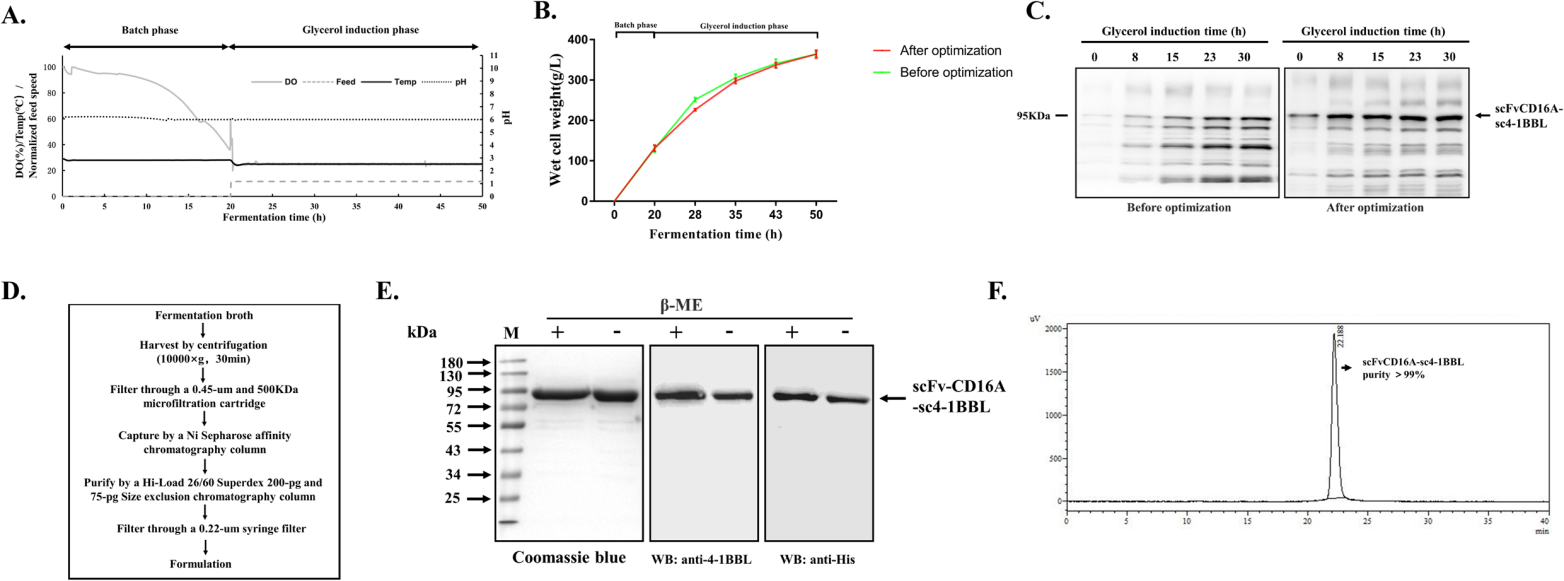
Pilot-scale fermentation, purification and characterization of scFvCD16A-sc4-1BBL.** a** Representative two-step fermentation process, including the batch phase and glycerol induction phase. Parameters, such as dissolved oxygen (DO), feeding speed, temperature and pH were monitored during fermentation. **b** Wet cell weight of scFvCD16A-sc4-1BBL-expressing strains before and after parameters optimization was monitored at indicated time points and represented as mean ± SD from 5 batches. **c** The expression of scFvCD16A-sc4-1BBL during fermentation was analysed by Western blotting using the same antibody as in Fig. 1c under nonreducing conditions. Left: before parameter optimization; right: after parameter optimization; **d** The downstream processing workflow of fermentation broth. **e** The purified scFvCD16A-sc4-1BBL was separated by SDS-PAGE under reducting or nonreducing conditions and identified by Coomassie blue staining or Western blotting. **f** The purity was determined with SEC-HPLC. M: prestained protein marker

We next optimized the manufacturing parameters of pilot-scale fermentation to acquire a high amount of recombinant protein with bioactivity. A two-step fermentation procedure (Fig. 2a), including a batch phase and a glycerol induction phase, was performed with optimized parameters, i.e., glycerol feeding rate, agitation speed and pH (Additional file 1: Table S1). After the batch phase, the wet cell weight (WCW) reached 131 ± 7.6 g/L, it increased gradually during the glycerol induction phase, and reached 364 ± 10.0 g/L at the end of fermentation (n = 5 batches, Fig. 2b). Though the WCW did not change significantly after parameter optimization, the yield and purity of scFvCD16A-sc4-1BBL in the supernatant were largely improved (Fig. 2c). At the end of the fermentation, the percentage of scFvCD16A-sc4-1BBL in the supernatant was approximately 40% in all forms of recombinant protein analyzed by densitometric measurement (Fig. 2c). The supernatant was collected by centrifugation and filtration, and then the complexes were enriched by Ni Sepharose affinity chromatography and further purified by HiLoad 26/60 Superdex 200-pg size and Hiload 26/60 Superdex 75-pg size exclusion chromatography. The recovery of scFvCD16A-sc4-1BBL after affinity and size exclusion chromatography was 23.66 ± 2.38% and 49.38 ± 3.57% (mean ± SD from 2 batches). And the final product was preserved in 1 × PBS (pH7.4) solution (Fig. 2d). The purified protein complex was analyzed through SDS-PAGE with Coomassie blue staining, Western blotting and HPLC (Fig. 2e and f). The results of SDS-PAGE and Western blotting showed apparently high purity of our target protein, which was consistent with the data of HPLC (above 99% purity).

### The two moieties of the scFvCD16A-sc4-1BBL complex retain their binding specificities

**Fig. 3:**
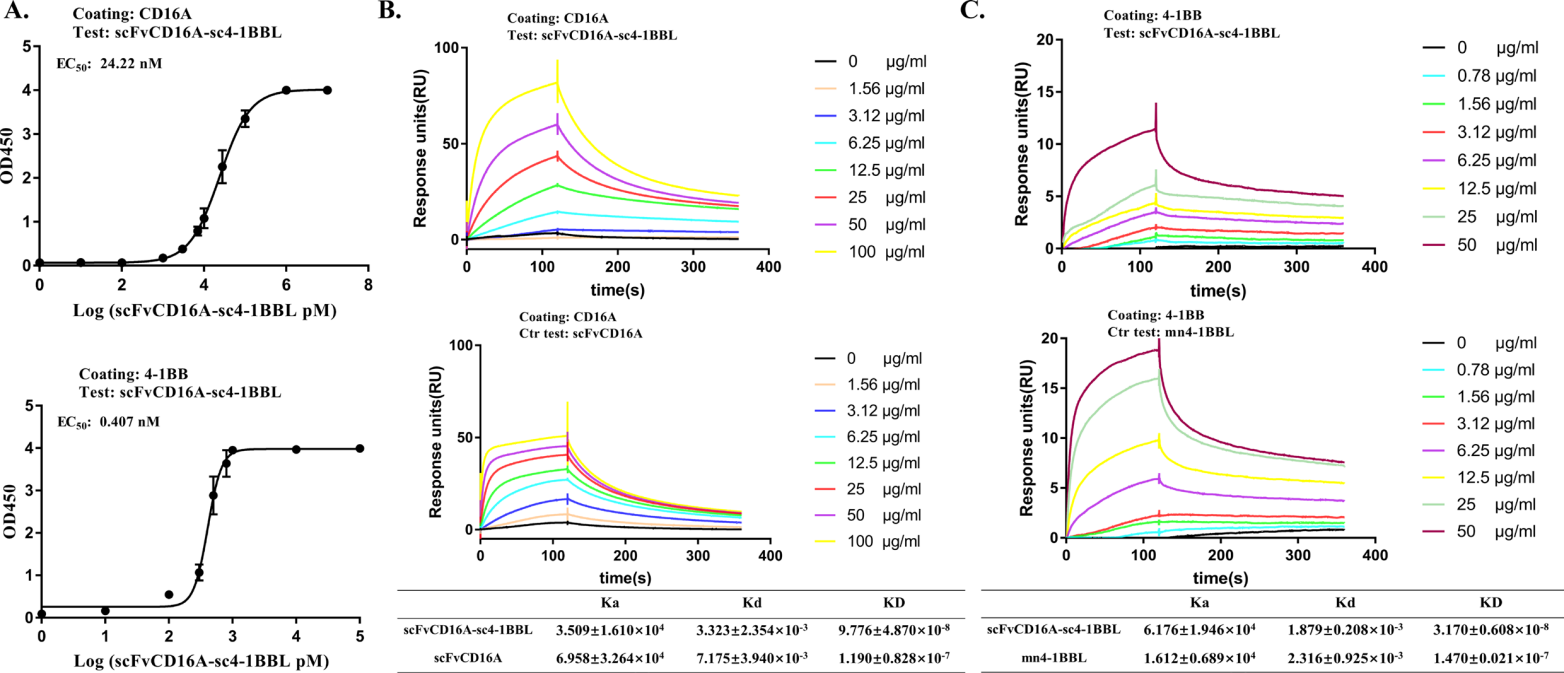
Binding properties of scFvCD16A-sc4-1BBL to its receptors. The affinity of scFvCD16A-sc4-1BBL to human CD16A and 4-1BB was analyzed by ELISA (**a**) and Surface Plasmon Resonance (SPR) (**b** and **c**). The concentrations of the tested proteins are indicated. **a.** The curves are ELISA results showing the binding of scFvCD16A-sc4-1BBL to human CD16A receptor (top panel) or human 4-1BB receptor (bottom panel), all the points are shown as the mean ± SD from three independent assays, n = 3. **b** The scFvCD16A-sc4-1BBL (top panel) or the reference protein scFvCD16A (bottom panel) binds to human CD16A, and the table shows the analysis results from the figure. **c.** The scFvCD16A-sc4-1BBL (top panel) or the control protein mn4-1BBL (bottom panel) binds to the human 4-1BB receptor, and the table shows the analysis results from the figure. Ka: association rate constant; Kd: dissociation rate constant; KD (= Kd/Ka): equilibrium dissociation (binding) rate constant; EC_50_: concentration to reach half of the maximal binding

First, we examined whether the two parts of the complex could bind with human CD16A and the 4-1BB receptor properly by ELISA and Surface Plasmon Resonance (SPR). ELISA results showed that the EC_50_ values of scFvCD16A-sc4-1BBL to bind with CD16A and 4-1BB were 24.22 nM and 0.407 nM, respectively (Fig. 3a), suggesting that the two moieties of the complex retain their individual binding specificities. We next compared the binding affinities of scFvCD16A-sc4-1BBL to human CD16A and 4-1BB receptors in parallel with its two individual moieties scFvCD16A and mn4-1BBL by SPR (Fig. 3b and c). The human CD16A and 4-1BB proteins were coupled with a CM5 chip, and then different concentrations of scFvCD16A-sc4-1BBL (or parental proteins) flowed through the chip. The equilibrium dissociation (binding) constants (KD) of scFvCD16A-sc4-1BBL to CD16A and 4-1BB were 97.76 nM and 31.70 nM, respectively (Fig. 3b and c), and those of their corresponding parental proteins to respective receptors (scFvCD16A to CD16A and mn4-1BBL to 4-1BB) were 119 nM and 147 nM. These KD values are all in the nM range and close to those of previously described anti-CD16A antibodies [32] and homotrimer 4-1BBL [51]. Collectively, these results indicated that the scFvCD16A-sc4-1BBL produced in *K. phaffii* can properly bind to human CD16A and 4-1BB.

### scFvCD16A-sc4-1BBL promotes the proliferation of PBMC-derived NK cells in vitro

**Fig. 4:**
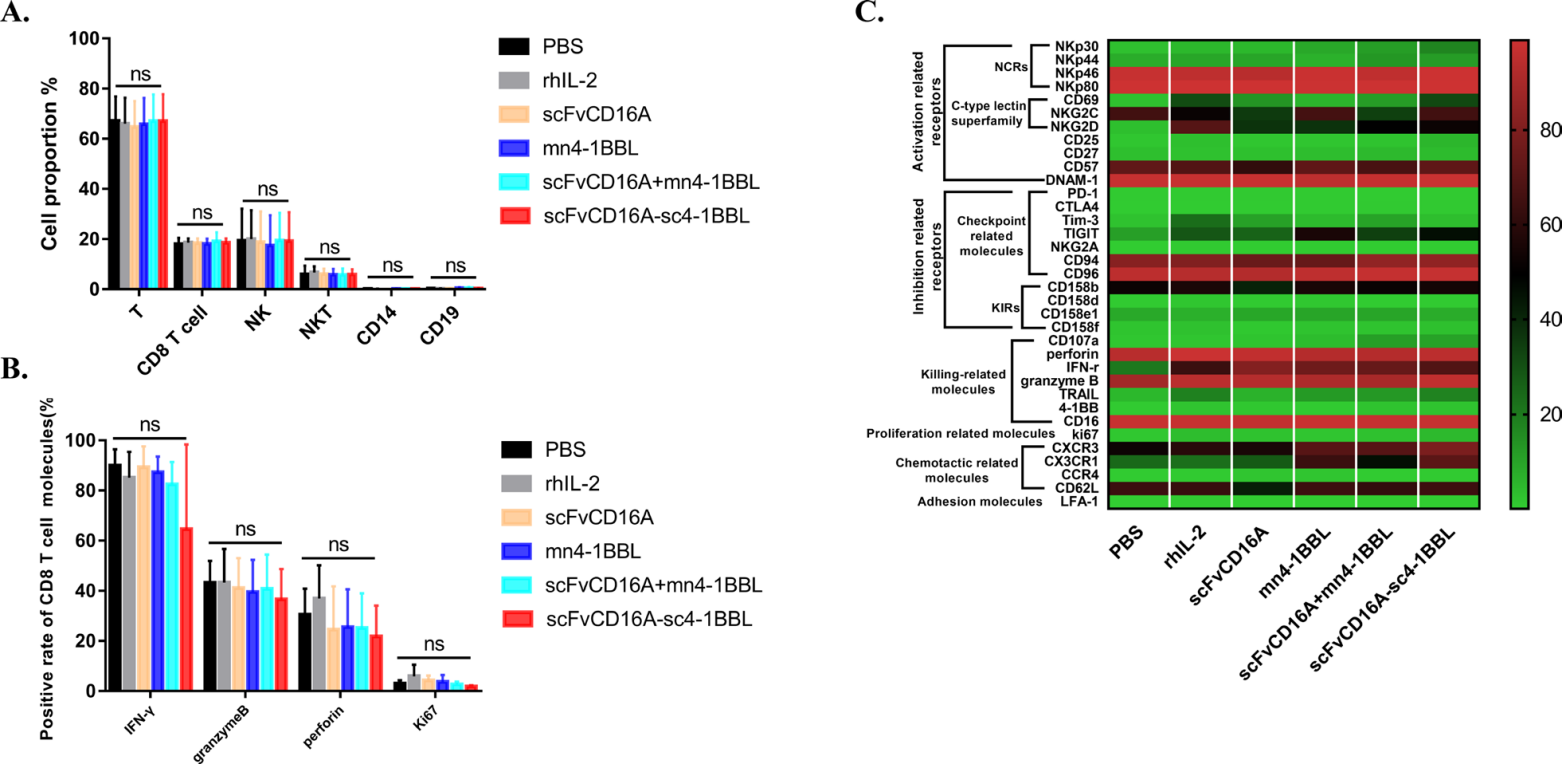
scFvCD16A-sc4-1BBL activated PBMC-derived NK cells. Human PBMCs from healthy donors (n = 5) were cultured in 1640 media for 24 h in the presence of 50 IU/mL rhIL-2, 20 nM scFvCD16A-sc4-1BBL, 20 nM scFvCD16A, 60 nM mn4-1BBL or 20 nM scFvCD16A + 60 nM mn4-1BBL, respectively. The expression of the indicated protein markers on NK cells (CD3^−^CD56^+^) and CD8 + T cells was analysed by flow cytometry. **a** Cell proportions of different immune cells under the indicated treatments. **b** The expression levels of IFN-γ, Granzyme, Perforin, Ki67 in CD8^+^ T cells. **c** Heatmap showing the expression levels of surface receptors and intracellular granule components in NK cells under the indicated treatments. Datas are represented as the mean ± SD from 5 independent experiments. ns: not significant; * *p* < 0.05; ** *p* < 0.01

**Fig. 5:**
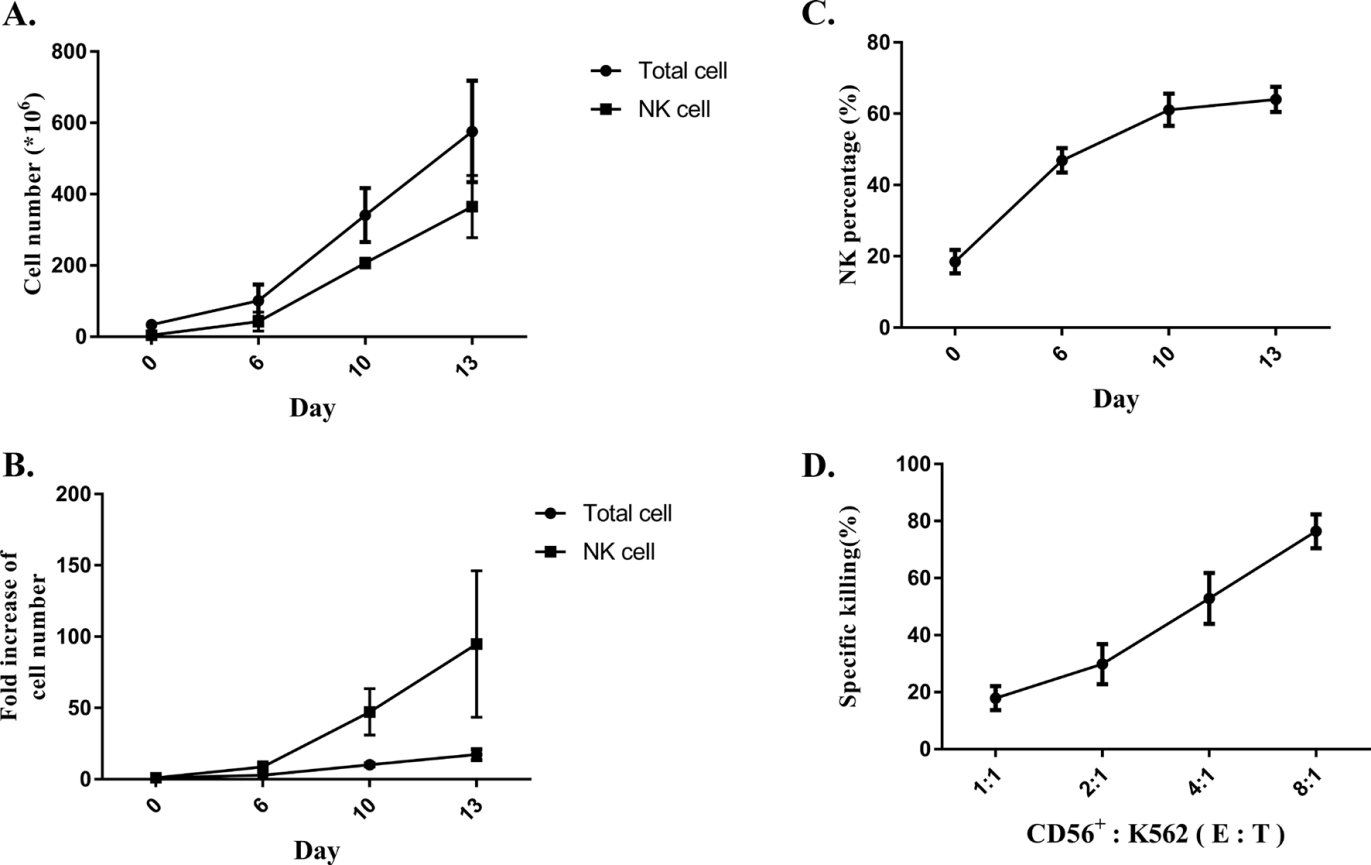
scFvCD16A-sc4-1BBL stimulated the proliferation of PBMC-derived NK cells, and the expanded NK cells were cytotoxic to K562 cells. 1.5 X 10^6^ PBMCs from healthy donors (n = 6) were cultured in KBM581 medium with 2 μg/ml scFvCD16A-sc4-1BBL precoated on the flask for 13 days and supplemented with rhIL-2 ( 1000 IU/ml). **a** The curves show the counts of NK cells and total cells. **b** The increased folds of NK cell number and total cell number. **c** The proportion of NK cells. **d** The cytotoxicity of expanded NK cells was analysed by a killing assay, and the curve shows the specific killing percentage of expanded NK cells targeting K562 cells at different E:T ratios. The data are shown as the mean ± SD from 6 independent experiments

To identify the biofunction of scFvCD16A-sc4-1BBL, we sought to characterize its selectivity to activate and expand NK cells from PBMCs. PBMCs were freshly isolated from healthy donors, and then treated with 50 IU/ml rhIL-2 or distinctive fusion proteins (i.e. 20 nM scFvCD16A-sc4-1BBL, 20 nM scFvCD16A, 60 nM mn4-1BBL, 20 nM scFvCD16A + 60 nM mn4-1BBL) for 24 h. PBS-treated cells served as a control. After a short period (24 h), the proportions of cell subsets in PBMCs and the specific marker of CD8^+^ T cells were not changed in any of the six conditions (Fig. 4a and b). In contrast, scFvCD16A-sc4-1BBL treatment significantly increased the expression levels of several surface receptors in NK cells, such as activation-related receptors (CD69, CD25, NKG2D and NKp30), killing-related molecules (4-1BB, CD107a, and TRAIL) and the proliferation-related molecule Ki67, compared to PBS treatment (Fig. 4c and Additional file 1: Fig. S5). These results illustrated that scFvCD16A-sc4-1BBL can directly stimulate NK cells among PBMCs.scFvCD16A-sc4-1BBL preferentially upregulates membrane markers of NK cells among PBMCs. Moreover, the proliferation-related molecule Ki67 increased in NK cells but not in T cells (Fig. 4). We subsequently explored whether this complex can stimulate the proliferation of NK cells in vitro and whether the cytotoxicity of the expanded NK cells can be conserved. Freshly isolated PBMCs were cultured for 13 days with scFvCD16A-sc4-1BBL immobilized on the bottom of the flasks in the presence of rhIL-2. As shown in Fig. 5a, the total cell number reached approximately 575 ± 142 × 10^6^ and the NK cell number reached 365 ± 87 × 10^6^. The fold changes in total cell and NK cell numbers after culture were 17.4 ± 3.7 and 95.9 ± 51.4 respectively (Fig. 5b), and the percentage of CD3^−^CD56^+^ NK cells reached 64.1% ± 8.7% (Fig. 5c). Importantly, expanded NK cells efficiently killed 76.4% ± 14.5% of K562 cells at the highest ratio of effector (notably CD56^+^ cells) and target cells (Fig. 5d), which is similar to the killing activity of expanded NK cells by the feeder-free protocol [29]. These results demonstrated that following scFvCD16A-sc4-1BBL treatment with rhIL-2, NK cells from PBMCs expand in vitro and the killing activity is conserved.

### scFvCD16A-sc4-1BBL enhances the antitumor activity of adoptively transferred NK cells in a xenograft murine ovarian cancer (OC) model

**Fig. 6:**
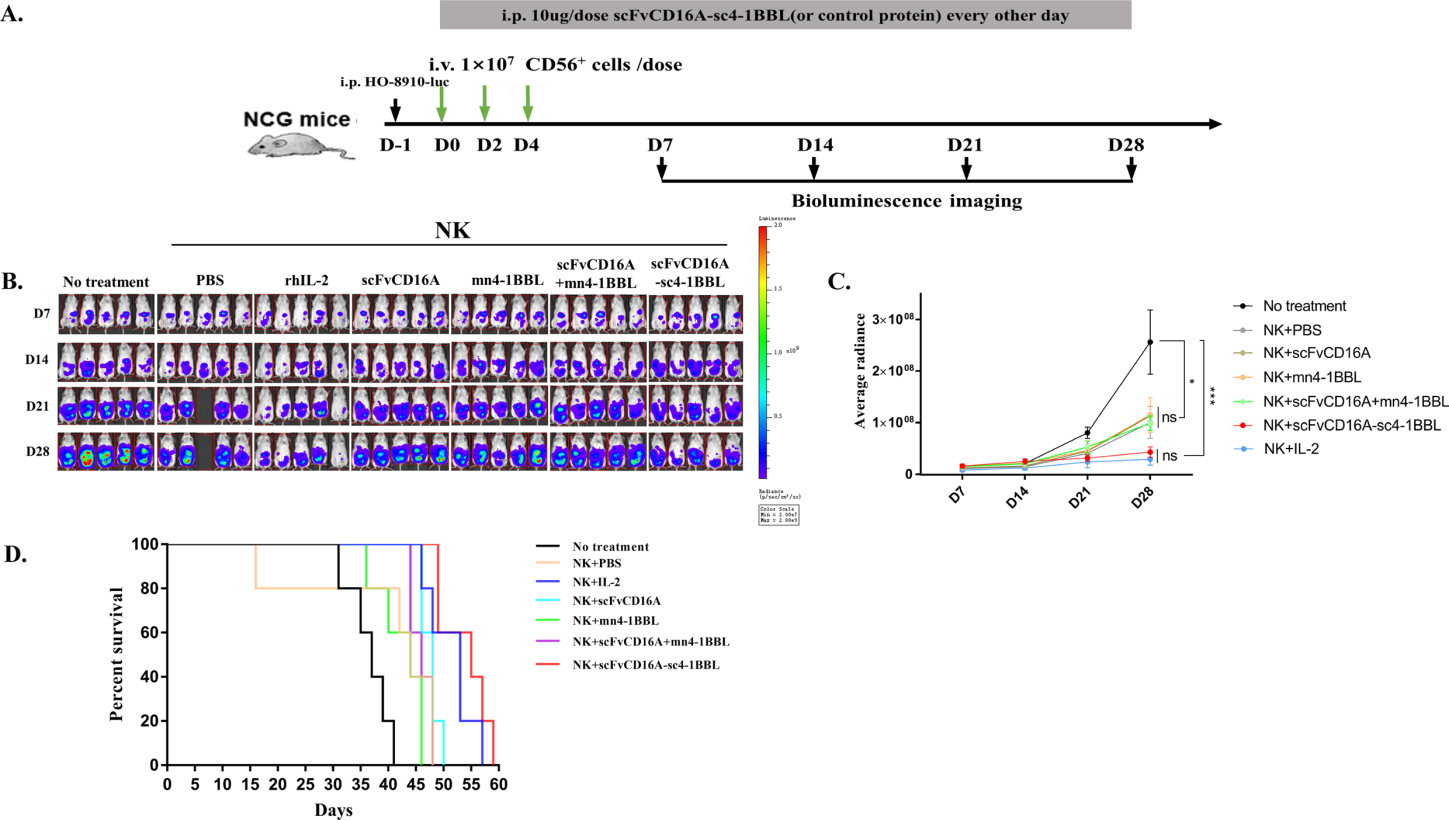
scFvCD16A-sc4-1BBL enhanced the therapeutic effect of adoptively transferred NK cells in a xenograft model of human epithelial ovarian cancer (OC).** a** The schematic timeline of the establishment of a human OC solid tumor model with Ho-8910 in NCG mice and treatments. **b** Animal bioluminescence imaging was used to quantify the tumor burden of different combination treatment groups on the indicated days. One mouse from the NK + PBS group died accidently **c** Summary of bioluminescence measurements for different combination treatment groups. **d** The survival curves of mice in different combination treatment groups. Data are represented as the mean ± SD, n = 5 mice per group. Significant differences were determined by one-way ANOVA. * *p* < 0.05; ** *p* < 0.01; *** *p* < 0.001

Our previous studies demonstrated that ex vivo expanded PBMC-derived NK cells by the defined feeder-free protocol effectively reduced the tumor burden and ascites in a xenograft murine OC model [29]. scFvCD16A-sc4-1BBL can stimulated the activation and expansion of NK cells from PBMCs in *vitro*. Thus, to what extent can scFvCD16A-sc4-1BBL stimulate adoptively transferred NK cells in vivo? We then proceeded to proof-of-concept experiments using the solid tumor model of human epithelial ovarian cancer by intraperitoneal injection of Ho-8910-Luciferase (Luc) cells into NCG mice (Fig. 6a). NK cells (notably 1X 10^7^ CD56^+^ cells) were administered in two-day intervals three times; moreover, scFvCD16A-sc4-1BBL or three types of parental moieties (i.e., scFvCD16A, mn4-1BBL and a combination of both (scFvCD16A + 3- fold of mn4-1BBL) were intraperitoneally injected every other day in parallel with the reference drug rhIL-2 (50,000 IU/mouse). Administration of PBS without NK cells was used as a control.

Tumor development was detected weekly with a bioluminescence imaging system (Fig. 6b and c). NK cell infusion reduced the growth of ovarian tumors (no treatment vs NK + PBS, * *p* < 0.05), and the addition of rhIL-2 injection to facilitate NK efficacy led to more significant inhibition of tumor growth (no treatment vs NK + IL-2, *** *p* < 0.001). Importantly, the combination of NK cell infusion and scFvCD16A-sc4-1BBL injection significantly inhibited the tumor burden (no treatment vs NK + scFvCD16A-sc4-1BBL, *** *p* < 0.001), and its efficacy in inhibiting tumor burden was comparable to the combination of NK and rhIL-2 (NK + scFvCD16A-sc4-1BBL vs NK + rhIL-2, ns *p* > 0.05). Notably, the combinations of NK cell infusion with each of the three types of parental moieties did not further reduce the tumor burden induced by NK cell infusion individually. Furthermore, the survival rate of the combinational treatments (NK + scFvCD16A-sc4-1BBL) was superior to that of all the other six treatments (Fig. 6d). The weight changes of mice under this condition during the whole experimental process were always less than 10%, without an obvious sign of toxicity (Additional file 1: Fig. S6). These results together illustrated that protein complex scFvCD16A-sc4-1BBL injection enhances the efficacy of NK cell infusion against solid ovarian tumor in a mouse model without observed toxicity.

## Discussion

In this study, we designed a novel antibody-ligand fusion protein containing the scFv of CD16A and the ECDs of 4-1BBL in the single-chain format and selected *K. phaffii* as the expression platform to produce this recombinant protein. Our investigations demonstrated that this fusion protein is capable of stimulating the activation and expansion of PBMC-derived NK cells in vitro, and enhancing the efficacy of adoptively transferred PBMC-derived NK cells in a mouse model of epithelial ovarian cancer.

Concerning pharmaceutical protein production, mammalian hosts have apparently outperformed microbial expression systems. In recent years, approximately 80% of recombinant therapeutic proteins have been expressed in mammalian expression system [47]. But the microbial system, especially yeast system, can be an alternative to produce pharmaceutical protein with improved folding machinery and less cost [52–54]. In this study, we employed *K. phaffii* as the expression production host, successfully expressed the antibody-ligand recombinant protein (Fig. 1), and subsequently established a pilot-scale protein cultivation and purification process, which allowed stable production of recombinant protein with high purity (Fig. 2) and biological activity (Figs. 3, 4, 5, 6).

The antitumor ability of NK cells is determined by the comprehensive effects of the activation and inhibitory molecules expressed on the surface of NK cells [4, 14, 55]. When NK cells are in a strong inhibitory tumor microenvironment, the inhibitory signals suppress NK cells and make them in a state of immune exhaustion. There are two major approaches to reversing the exhaustion of NK cells. One is to block the inhibitory signal to relieve the immune exhaustion of NK cells, e.g., antibodies blocking the PD-1/PD-L1 immune checkpoint. The second is to provide NK cells with activation signals to counteract the immunosuppressive effect caused by the inhibitory signal. [14, 15, 18]. CD16A is the principal activating molecule of NK cells and is currently the main target for the development of drugs to enhance NK cell function. Several CD16A-based drugs have been developed to enhance the antitumor activity of NK cells, including monoclonal antibodies and bispecific antibodies [16, 21, 56–58]. In this study, we selected the single-chain variable fragment of the human CD16A antibody, aiming to target and enhance the function of NK cells. Considering that a single CD16A signal may not be sufficient to boost the biofunction of NK cells, we also selected an additional stimulatory molecule, 4-1BB, to induce a dual activation signal in NK cells. 4-1BB provides strong costimulatory activity for T cells and enhances the antitumor activity and proliferation ability of T cells [59]. The activation of the 4-1BB receptor by membrane-bound 4-1BBL can also promote the proliferation ability of NK cells and enhance the antitumor activity of NK cells in vitro [38, 39, 60]. As shown in Fig. 4 and Additional file 1: Fig. S5, the scFvCD16A-sc4-1BBL complex significantly upregulated activation receptors (CD69, CD25, NKG2D and NKp30), killing-related molecules (4-1BB, CD107a, and TRAIL) and the proliferation-related molecule Ki67 of NK cells, which were not observed in the cells treated with scfv-CD16A, mn4-1BBL or the combination of them.

An ideal scenario for scFvCD16A-sc4-1BBL to exert its biofunction in vivo would be as follows: it selectively binds to CD16A-positive NK cells with the moiety of scFv CD16A, and another moiety, ECDs of 4-1BBL, binds with the 4-1BB receptors of NK cells; hence, dual activation signalling on NK cells is guaranteed. Considering that the 4-1BB receptor is widely expressed in a variety of immune cells, scFvCD16A-sc4-1BBL may also regulate other immune cells in vivo, e.g., T cells. We additionally examined the expression of several essential functional molecules (i.e., IFN-γ, Granzyme, Perforin, Ki67) on CD8^+^ T cells from PBMC, following the scFvCD16A-sc4-1BBL treatment, but we did not observe a bias effect of the protein complex on T cells (Fig. 4b). Moreover, scFvCD16A-sc4-1BBL specifically stimulates the proliferation of NK cells, rather than T cells or other immune cells among PBMCs (Fig. 5).

Acquiring a high quantity of PBMC-derived NK cells to meet clinical requirements has always been one of the hurdles limiting the application of adoptive NK cell transfer therapy. Liu et al. used three cytokines (IL-2, IL-15 and IL-18) to expand PBMC-derived NK cells; the purity of NK cells reached 71%, and the number of NK cells increased by 294 folds after 14 days of culture [61]. Choi et al. used a combination of several cytokines, including IL-2, IL-15 and IL-18, as well as antibodies against CD16, CD56 and NKp46 to expand PBMC-derived NK cells. After 14 days of culture, the purity of NK cells reached to 64.7% ± 9.6%, and the fold increase in NK cells reached a maximum 140-fold [30]. In this study, scFvCD16A-sc4-1BBL stimulated the activation and proliferation of PBMC-derived NK cells in vitro to the extent that it was not inferior to NK cells following other cytokines and/or antibody-based protocols. scFvCD16A-sc4-1BBL was capable of improving the purity of PBMC-derived NK cells to 64.1 ± 8.7%(Fig. 5b), and the NK cell number increased 95.9 ± 51.4-fold (Fig. 5c) after 13 days of culture. Importantly, the in vitro proliferated NK cells retained their cytotoxicity against K562 cells (Fig. 5d). Collectively, scFvCD16A-sc4-1BBL can be an alternative stimulator for PBMC-derived NK cell expansion in vitro.

Ovarian cancer is one of the most malignant tumors, and its 5-year survival rate for patients with advanced ovarian cancer is only 6% [62, 63]. Approximately 60–80% of ovarian cancer patients achieve complete remission through surgery or chemotherapy, but 60% of them will relapse within 3 years, and more than half of the patients with recurrence have chemotherapy resistance [63–65]. Therefore, exploring more effective treatments is urgently needed. Emerging immunotherapies, such as tumor vaccines, immune checkpoint blockade, and CAR-T therapy, have brought new hope to the treatment of ovarian cancer. However, due to the lack of effective antigenic targets, these immunotherapies have not achieved the desired results [63, 66]. Adoptive transfer of NK cells can recognize and kill tumor cells without MHC restriction, so it may be a better option to drive NK cells to treat ovarian cancer [63, 67, 68]. Previous studies in our laboratory showed that the adoptive transfer of PBMC-derived NK cells expanded by a feeder-free protocol combined with rhIL-2 effectively inhibited the growth of ovarian tumors in mice [29]. Here, we utilized this model and illustrated that scFvCD16A-sc4-1BBL enhanced the anti-solid tumor effect of NK cells without observed toxicity. In particularly, only scFvCD16A-sc4-1BBL, rather than its individual parental moieties, additionally reduced the tumor burden and prolonged mouse survival to the extent that only the combination of NK adoptive transfer and rhIL-2 injection reached. The improved efficacy in vivo follows several possible mechanisms, such as further proliferation and activation of NK cells, extended lifespan of NK cells and enhanced infiltration and persistence in the tumor microenvironments (TME), which deserve further investigation.

IL-2 has long been used to activate immune cells. It was approved by the FDA for the treatment of renal cell carcinoma and metastatic melanoma [69, 70]. However, IL-2 application revealed limited efficacy due to its short half-life and severe toxicity [69]. IL-15 is another potent stimulator for NK cells and CD8^+^ T cells with a safer profile but unsatisfactory efficacy in vivo [71]. Notably, both cytokines inclusively activate a series of immune cells, not only NK cells. Our antibody-ligand fusion protein was designed to direct NK cells by targeting their specific activating receptor CD16, accompanied by targeting costimulatory receptor 4-1BB, which could lead to specific, or at least, preferentially activation of NK cells.

## Conclusion

In this study, we demonstrated the feasibility of expressing a recombinant protein scFvCD16A-sc4-1BBL in *K. phaffii* with favourable biological activity. scFvCD16A-sc4-1BBL stimulated the expansion of PBMC-derived NK cells in vitro with retained cytotoxicity. Moreover, scFvCD16A-sc4-1BBL enhanced the therapeutic efficacy of adoptively transferred NK cells in a xenograft epithelial ovarian cancer model. We provide evidence that the expression of scFvCD16A-sc4-1BBL in *K. phaffii* holds translational value as a stimulator of NK cells.

## Materials and methods

### Strains, plasmids and antibodies

The *K. phaffii* strain X33 and *Escherichia coli* strain TOP10 were purchased from Invitrogen (Carlsbad, CA). The expression vector pGAPzα-rDNA was constructed and preserved in our lab. HRP conjugated-anti-His-antibody (A00612) was purchased from Genscript.

### Mice and cell lines

Female NCG (NOD/ShiLtJGpt-Prkdc^em26Cd52^Il2rg^em26Cd22^/Gpt) mice were purchased from Jiangsu Gempharmatech. NCG mice were maintained in accordance with the guidelines for laboratory animals approved by the Animal Research Committee of the University of Science and Technology of China and housed in an ultraclean barrier facility. The K562 (RRID:CVCL_0004) cell line was purchased from the Cell Bank of the Chinese Academy of Sciences (Shanghai, China). The Ho8910-Luc cell line was constructed and cryopreserved in our laboratory [29]. All cell lines were maintained in modified RPMI medium (HyClone, SH30809.01) supplemented with 10% FBS (Biological Industries, Beit HaEmek, Israel), 100 U/mL penicillin and 100 µg/mL streptomycin (Sangon Biotech, Shanghai, China) at 37 °C in a 5% CO_2_ incubator. All the cell lines were identified with STR profiling by Genewiz cpmpany (Suzhou, China).

### Molecular design and construction of expression vectors

The sequence of scFvCD16A was obtained from previous studies [32], and the sequence of the extracellular domain (71–254) of 4-1BBL was obtained from NCBI. The scFvCD16A-sc4-1BBL fusion gene was constructed by linking scFvCD16A and 4-1BBL with GS Linker. Codon optimization was performed, and restriction sites for enzymes Xhol and Not I were added at both ends of the fusion gene. The whole sequence was synthesized (Genewiz) and inserted into the Zeocin- resistant vector pGAPzα-rDNA, and the protein expression vector pGAPzα-rDNA-scFvCD16A-sc4-1BBL was constructed. Accordingly, the expression vectors of the single moieties pGAPzα-rDNA-scfv-CD16A and pGAPzα-rDNA-mn4-1BBL were constructed. (Additional file 1: Figs. S1 and S3).

### Screening for expression

The expression vector pGAPzα-rDNA-scFvCD16A-sc4-1BBL was linearized by the restriction enzyme Spe I, and electroporated into the *K. phaffii* X33 strain, and then the cell suspension was spread on YPD plates containing 300 µg/ml zeocin (Sigma). The protein expression level of the positive clones was detected with dot blotting. The selected clones with high expression were further identified by western blotting, and the clone with the highest expression was selected for subsequent pilot-scale fermentation. The control protein was screened in the same way (Additional file 1: Figs. S1 and S3).

### Fermentation and purification

A two-step fermentation was performed using a 14-L NBS BioFlo fermenter (Eppendorf, Hamburg, Germany) according to Invitrogen’s Pichia Fermentation Process Guidelines and our previous work [71]. Briefly, 400 ml of *K. phaffii* seed culture was added to a fermenter containing 6 L of BMGY medium containing 4% (w/v) glycerol to initiate fermentation. In the basal glycerol culture stage, the temperature was set at 28 ℃, and the pH was maintained at 6.0 by adding ammonia hydroxide. During the glycerol induction phase, 50% (w/_V_) glycerol feed containing 12 mL/L PTM1 solution was constantly added into the fermenter (feeding rate: 70 mL/h) to induce the synthesis of recombinant protein. Moreover, the dissolved oxygen (DO) was set to approximately 25% of air saturation, the stirring speed was 1000 rpm, the culture temperature was set to 25 °C and the glycerol induction phase time was restricted to no more than 30 h to avoid the degradation of target products. When the fermentation process was over, the fermentation supernatant was collected by centrifugation for 30 min at 10,000 g and immediately filtered through 0.45-μm and 500-kDa microfiltration cartridges using the FlexStand system (GE Healthcare Life Sciences, Chicago, IL) immediately. Prior to affinity chromatography, the pH of the filtered supernatant was adjusted with ammonia hydroxide to 7.2 to 7.4. A Ni Sepharose affinity chromatography column was used to capture the target proteins from the supernatant, and a HiLoad 26/600 Superdex 200-pg size and a HiLoad 26/600 Superdex 75-pg size exclusion chromatography column were used to further purify proteins using the AKTA Pure25 System (GE Healthcare Life Sciences). The purity of the target protein was analysed with SDS-PAGE and HPLC. The endotoxin levels in the final products were measured using Tachypiens Amebocyte Lysate (TAL) purchased from Bioendo (Fujian, China) according to the manufacturer’s instructions. The bacterial endotoxin levels of the final purified scFvCD16A-sc4-1BBL complexes were below 0.01 EU/μg protein. The purified protein complexes were then filtered through 0.22-μm filters, aliquoted and stored at − 80 °C. The individual moiety protein was produced with the same protocol (Additional file 1: Figs. S2 and S4).

### Measurement of binding ability by ELISA

Human CD16A (R&D, 0425-FC) or 4-1BB (Genscript, Z03382) (1 μg/ml) was coated on Corning 96-well plates at 4 ℃ overnight, washed once with PBST, and then blocked with 5% BSA in PBS for 1 h. The plate was washed with PBST once followed by adding different concentrations of scFvCD16A-sc4-1BBL, and then incubated at room temperature for 2 h. The samples were washed three times with PBST, and then HRP-conjugated anti-His secondary antibody was added and incubated for 1 h at room temperature in the dark. The plate was washed with PBST five times, developer solution was added and incubated at room temperature for 15 min in the dark, and then 10% sulfuric acid was added and immediately placed on the OD450 reading plate. The obtained data were analysed using GraphPad Prism.

### Measurement of binding affinity by Surface Plasmon Resonance (SPR)

The binding ability of the recombinant protein scFvCD16A-sc4-1BBL to human CD16A and 4-1BB was evaluated by a Biacore T200 BioSensor (GE Healthcare) [72]. A CM5 sensor chip (GE Healthcare) was coated with human CD16A or 4-1BB protein (~ 100 resonance units (RU)). The contact time was set to 2 min at a flow rate of 30 μL/min, followed by dissociation for 2 min. The chip was regenerated through injections of glycine (pH 2.0) twice. Kinetic analysis was performed with Biacore T200 evaluation software v 2.0.1.

### PBMC isolation and culture in vitro

PBMCs were isolated from the peripheral blood of healthy blood donors by Ficoll-Hypaque (Solarbio, Beijing, China) density gradient centrifugation with informed consent at Anhui Blood Center. PBMCs were cultured in RPMI medium supplemented with 10% FBS in the presence of 50 IU/mL rhIL-2, 20 nM scFvCD16A-sc4-1BBL, 20 nM scFvCD16A, 60 nM mn4-1BBL, or 20 nM scFvCD16A + 60 nM mn4-1BBL for 24 h. The cells were collected and analysed by flow cytometry according to a previously described method [71].

### NK cell expansion in vitro

Purified PBMCs, at a density of 1.5 × 10^6^ cells/mL, were seeded in a flask (Corning, 430,168) coated with scFvCD16A-sc4-1BBL (2 μg/ml, 24 h at 4 °C), and cultured in KBM-581 medium (Corning, 88–51-CM) containing 5% heat-inactivated autologous plasma and 1000 IU/mL rhIL-2 (Jinsili, Jiangsu, China) for 13 days at 5% CO_2_ and 37℃, by adding fresh medium every 2–3 days to maintain the cell density between 1.3 and 3.2 × 10^6^ cells/mL. Total viable cells were counted using an automatic cell counter, Countstar, (Alit, Shanghai, China) with trypan blue. The percentages of NK cells (CD3^+^ and CD56^+^) were analysed by flow cytometry (Beckman).

### Cytotoxicity assay

First, 10^6^ K562 cells were collected and suspended in RPMI 1640 containing 1% fetal bovine serum and then incubated with 5 μM CFSE (Biolegend, San Diego, CA, USA, 423,801) at 37 ℃ for 15 min in the dark. Cells were washed three times and resuspended in RPMI 1640 medium containing 10% FBS. In Paraller, the expanded NK cells were collected and purified with anti-CD56 beads (Miltenyi), as desribed previously [29]. They were subsequently suspended in RPMI 1640 containing 10% FBS at the indicated density and incubated with K562 cells for 3.5 h at 37 ℃. Propidium iodide solution (Biolegend, 421,301) was added for 5 min at room temperature to label dead cells. The specific killing of expanded NK cells to K562 cells was analysed by flow cytometry (Beckman).

### Tumor mouse models

In vivo experiments were conducted in strict accordance with the guidelines of the Animal Research Committee of the University of Science and Technology of China. The female NCG mice used in this study were 8 to 10 weeks old. All mice were tumor-bearing by intraperitoneal injection of 8 × 10^5^ HO-8910-Luc cells/mice on the indicated days, and were randomly divided into several experimental groups (each group had five mice) for subsequent experiments [29]. The expanded NK cells were injected through the tail vein, and the combination therapy drug was administered by intraperitoneal injection as same as the route of IL-2, also a commonly used administration route for OC caner murine model [29, 73–75]. The experimental group was divided into the following groups: No treatment: tumor bearing only, no NK cells; NK + PBS: only NK cells infused; NK + rhIL-2: NK cells infused, and an additional 50,000 IU rhIL-2/mice injected intraperitoneally every two days. NK + scFvCD16A group: NK cells were infused, and 3.4 µg/dose/mouse scFvCD16A was administered as rhIL-2. NK + mn4-1BBL group: NK cells were infused, and 6.6 µg/dose/mouse mn4-1BBL was administered as rhIL-2. NK + scFvCD16A + mn4-1BBL group: NK cells were infused, and 3.4 µg/dose/mouse scFvCD16A and 6.6 µg/dose/mouse mn4-1BBL were administered as rhIL-2. NK + scFvCD16A-sc4-1BBL group: NK cells were reinfused, and 10 µg/dose/mouse scFvCD16A-sc4-1BBL was administered as rhIL-2.

To monitor tumor burden, mice were injected intraperitoneally with D-luciferin (150 µg/g) in PBS and imaged using the bioluminescence imaging system (IVIS Spectrum, PerkinElmer, Boston, MA, USA) according to the manufacturer’s instructions. Treatment effects were monitored by imaging on days 7, 14, 21 and 28.

### Statistical analysis

GraphPad Prism 7.0 software was used to perform statistical analysis in this study. The statistical significance was determined by one-way analysis of variance (ANOVA). The survival curve was analysed using the log-rank (Mantel–Cox) test. Data are presented as the means ± SDs (standard deviation). ns: not significant; *: *p* < 0.05; **: *p* < 0.01; ***: *p* < 0.001.

## Supplementary Information

**Additional file 1: Fig. S1.** Construction and expression of scFvCD16A, corresponding to Fig. 1. **Fig. S2.** Pilot-scale fermentation, purification and characterization of scFvCD16A, corresponding to Fig. 2. **Fig. S3.** Construction and expression of mn4-1BBL, corresponding to Fig. 1. **Fig. S4.** Pilot-scale fermentation, purification and characterization of mn4-1BBL, corresponding to Fig. 2. **Fig. S5.** The expression level of NK surface molecules after stimulated by scFvCD16A-sc4-1BBL, corresponding to Fig. 4. **Fig. S6.** Monitoring curve of weight change ratio in mice during the in vivo activity assay, corresponding to Fig. 6. **Table S1.** The optimized parameters of culture condition for batch fermentation.

## Data Availability

The datasets used and/or analysed during the current study are included in this article and its additional files, and are available from the corresponding author upon reasonable request.

## Abbreviations

NK: Natural killer
scFv: Single chain fragment variable
ECD: Extracellular domain
4-1BBL: 4-1BB ligand
GS linker: Gly-Ser linker
*K. phaffii*: *Pichia pastoris*
PBMC: Peripheral blood mononuclear cells
MHC: Major histocompatibility complex
ADCC: Antibody dependent cell medicated cytotoxicity
WCW: Wet cell weight
CAR-T: Chimeric Antigen Receptor T-Cell Immunotherapy
PBS: Phosphate buffered saline
rhIL-21: Recombinant human IL-21
mn4-1BBL: Monomer 4-1BBL
sc4-1BBL: Single chain of 4-1BBL
