# Supplementary material for: Development of an antibody-ligand fusion protein scFvCD16A-sc4-1BBL in Komagataella phaffii with stimulatory activity for Natural Killer cells
Source: Microb Cell Fact. 2023 Apr 11;22:67. doi: 10.1186/s12934-023-02082-6 (PMC10091686; doi:10.1186/s12934-023-02082-6)
Supplement: Supplementary file 1 — Additional file 1: Fig. S1. Construction and expression of scFvCD16A, corresponding to Fig. 1. Fig. S2. Pilot-scale fermentation, purification and characterization of scFvCD16A, corresponding to Fig. 2. Fig. S3. Construction and expression of mn4-1BBL, corresponding to Fig. 1. Fig. S4. Pilot-scale fermentation, purification and characterization of mn4-1BBL, corresponding to Fig. 2. Fig. S5. The expression level of NK surface molecules after stimulated by scFvCD16A-sc4-1BBL, corresponding to Fig. 4. Fig. S6. Monitoring curve of weight change ratio in mice during the in vivo activity assay, corresponding to Fig. 6. Table S1. The optimized parameters of culture condition for batch fermentation. [file 12934_2023_2082_MOESM1_ESM.doc]

**
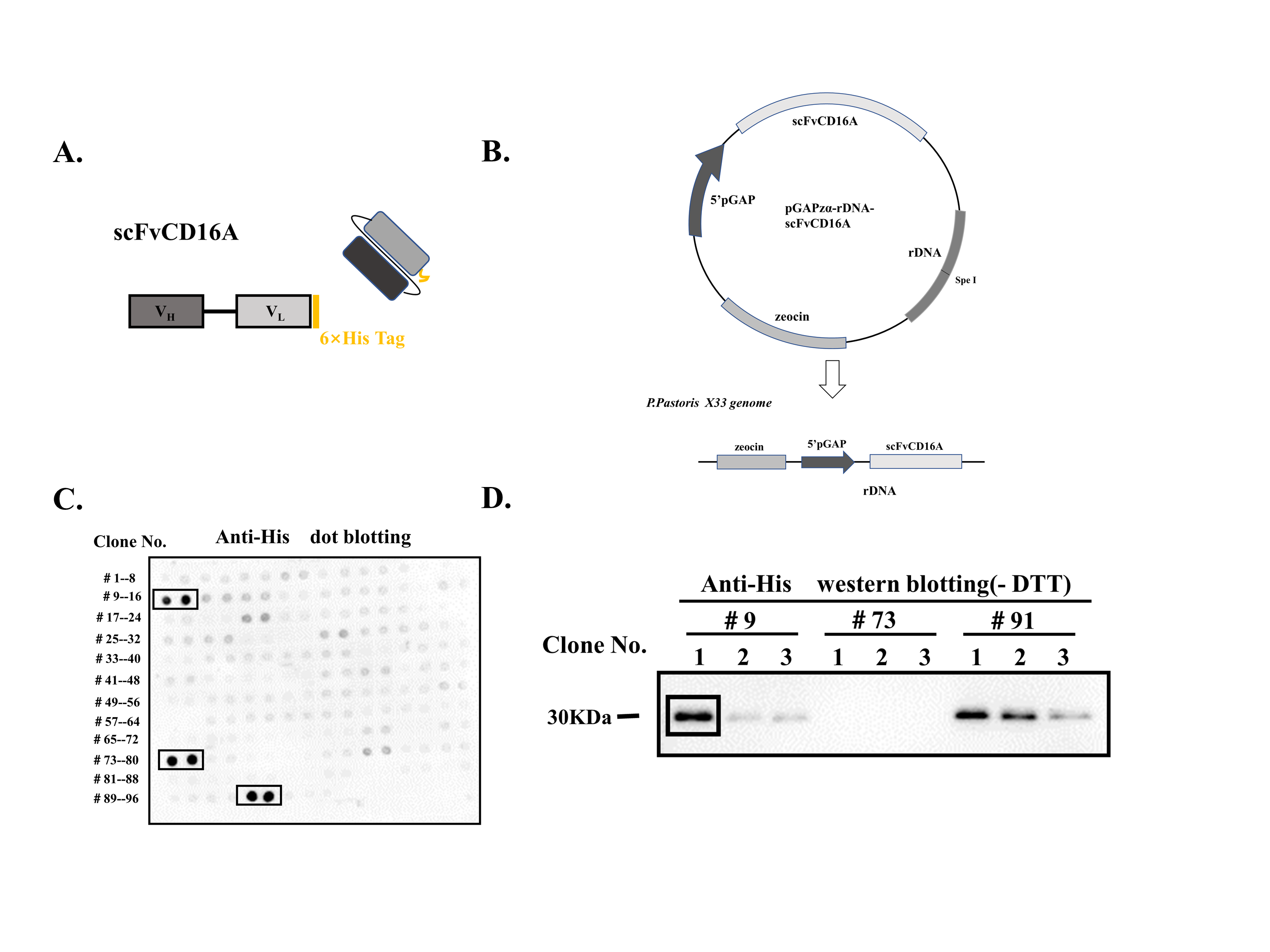
**

**Supplementary Fig 1. Construction and expression of scFvCD16A, corresponding to Fig 1. a.** Schematic diagram of the molecular structure and gene sequence of scFvCD16A. scFvCD16A was composed of a single chain human CD16A antibody fragment, with a 6X His tag at the C-terminal. **b.** The coding sequence for scFvCD16A was inserted into the pGAPzα-rDNA plasmid under the control of the GAP promoter, which then integrated the scFvCD16A codon cassette into the genome of *Pichia pastoris* X33 through homologous recombination. **c.** The positive clones expressing scFvCD16A were revealed by dot blotting using an anti-His tag HRP conjugate antibody. **d.** The high expression clones were further identified by western blotting under nonreducing conditions (-DTT) using the same antibody as c.. The clone of No. # 9-1 showed the highest expression and was selected for the following study.

**
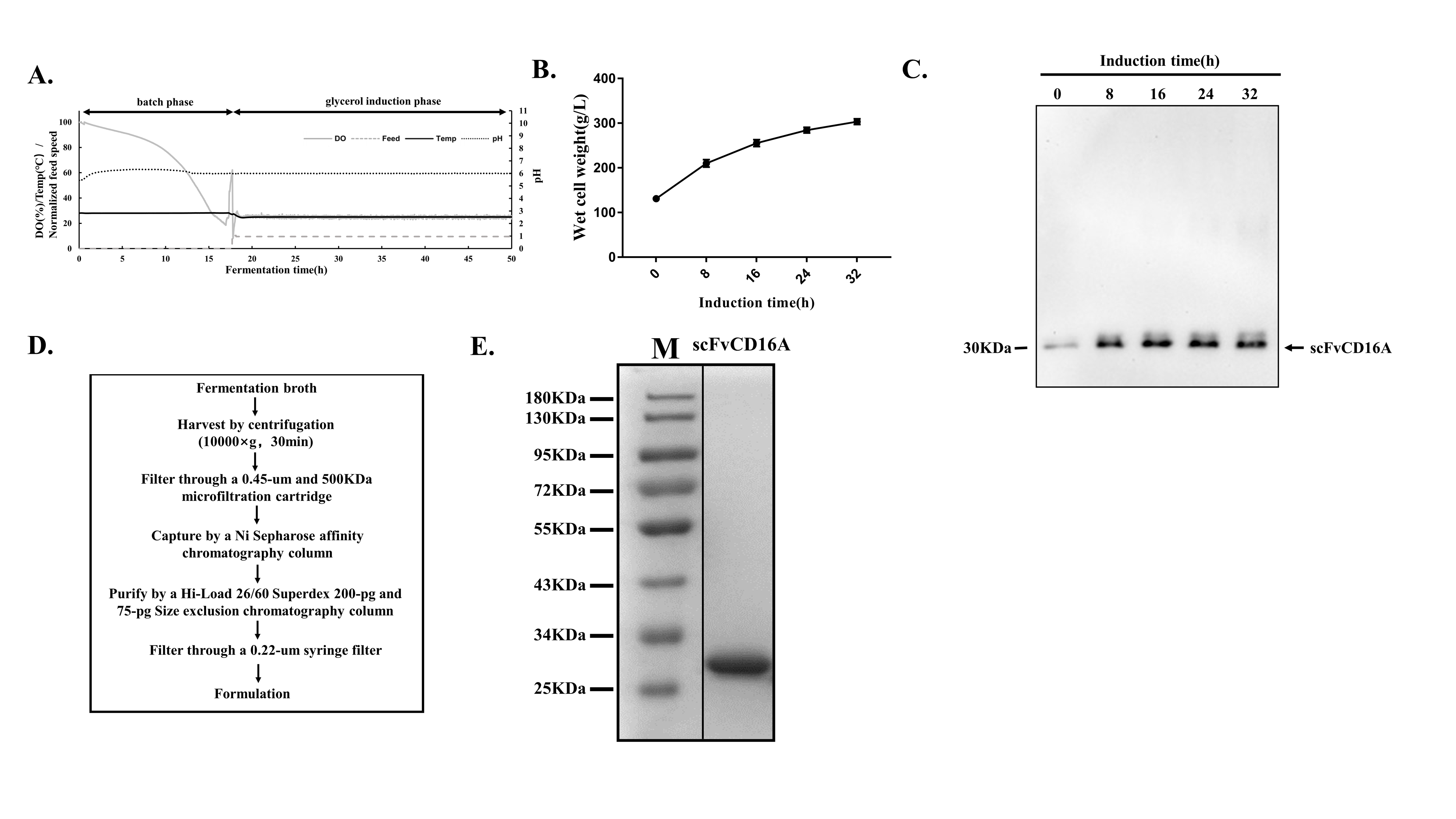
**

**Supplementary Fig 2. Pilot-scale fermentation, purification and characterization of scFvCD16A, corrresponding to Fig 2. a.** Representative two-step fermentation process, including the batch phase and glycerol induction phase. Parameters such as dissolved oxygen (DO), feeding speed, temperature and pH, were monitored during fermentation. **b.** Cell growth of scFvCD16A expressing strains during fermentation was monitored and represented as wet cell weight(n=5). **c.** The expression of scFvCD16A during fermentation was analyzed by Western blotting using an anti-His HRP conjugate under nonreducing conditions. **d.** The downstream processing workflow of fermentation broth. **e.** The purified scFvCD16A was separated by SDS-PAGE under nonreducing conditions and identified by Coomassie blue staining. M: prestained protein marker.

**
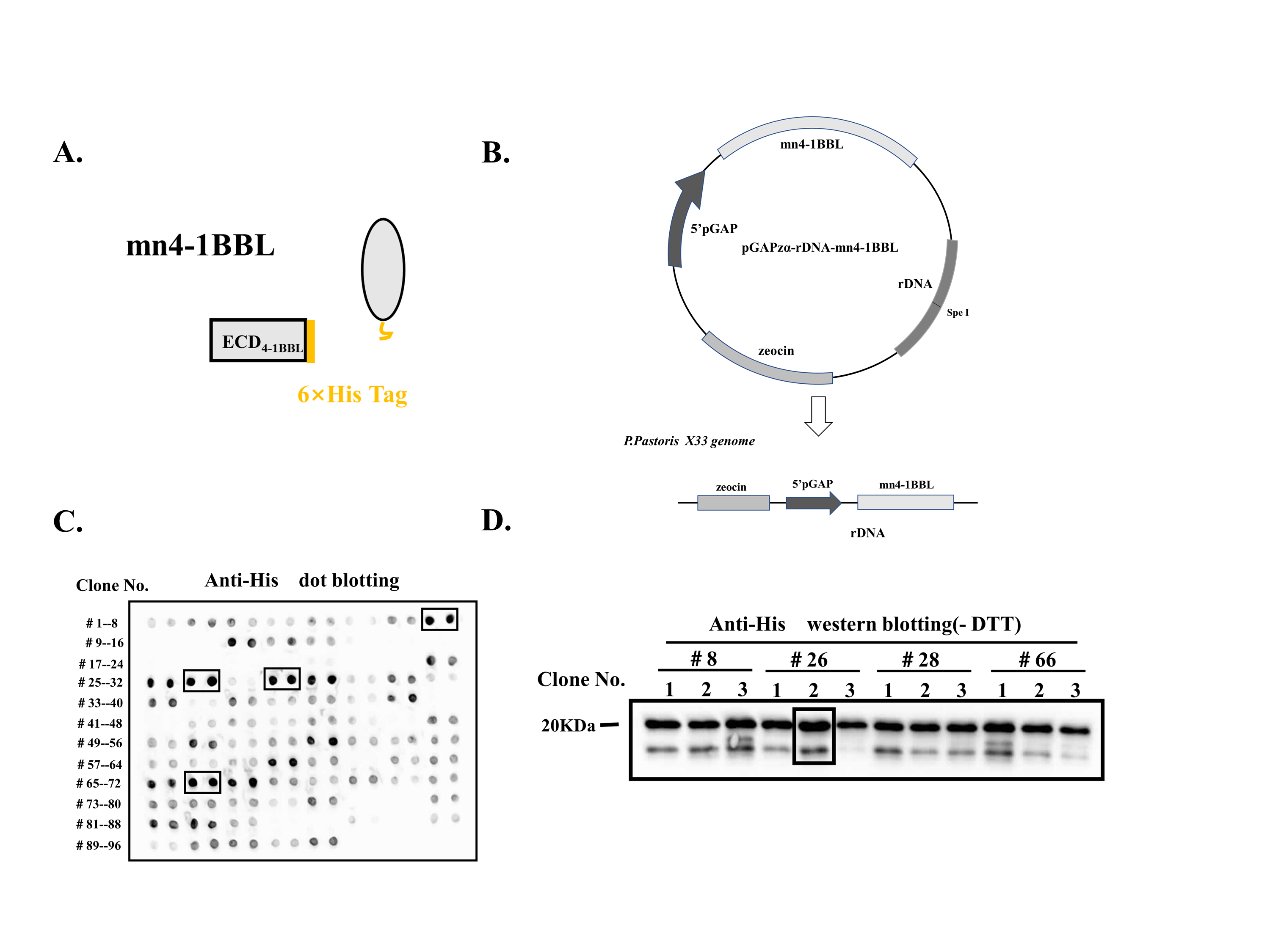
**

**Supplementary Fig 3 Construction and expression of mn4-1BBL, corresponding to Fig 1. a.** Schematic diagram of the molecular structure and gene sequence of mn4-1BBL. mn4-1BBL was composed of one extracellular domain (ECD) of 4-1BB ligand and a 6X His tag. **b.** The coding sequence for mn4-1BBL was inserted into the pGAPzα-rDNA plasmid under the control of the GAP promoter, which can integrate the mn4-1BBL codon cassette into the genome of *Pichia pastoris* X33 through homologous recombination. **c.** The positive colonies expressing mn4-1BBL were selected by dot blotting using an anti-His tag HRP conjugate antibody. **d.** The high expression clones were further selected and confirmed by Western blotting under nonreducing conditions using an anti-His tag HRP conjugate antibody. The clone of No. 26-2 was selected and used in this study.

**
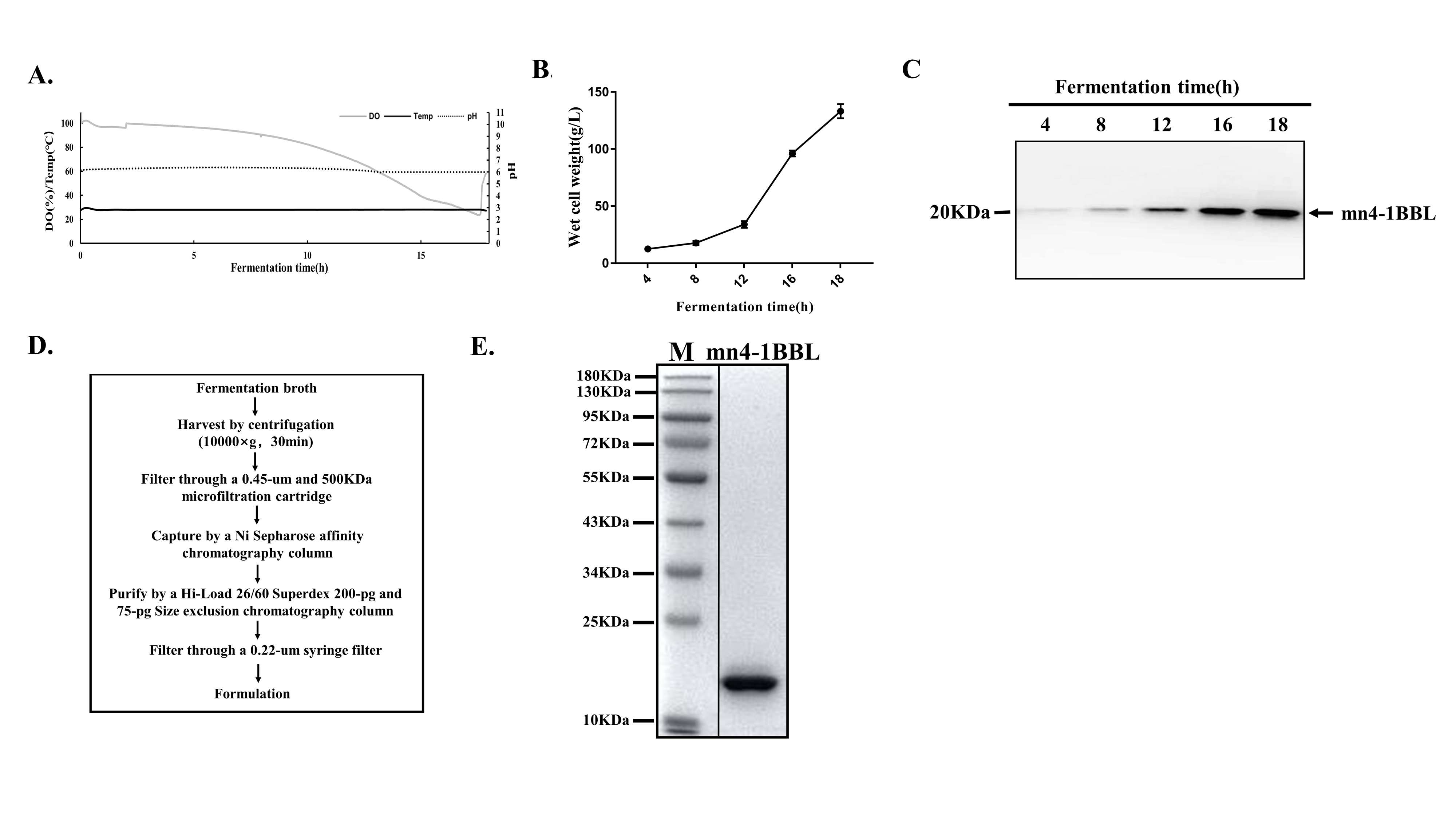
**

**Supplementary Fig 4. Pilot-scale fermentation, purification and characterization of mn4-1BBL, corresponding to Fig 2. a.** Representative fermentation process of pGAPzα-rDNA-mn4-1BBL X33 expressing strains. Parameters, such as dissolved oxygen (DO), temperature and pH, were monitored during fermentation. **b.** Cell growth of mn4-1BBL expressing strains during fermentation was monitored and represented as wet cell weight(n=5). **c.** The expression of mn4-1BBL during fermentation was analyzed by Western blotting using an anti-His HRP conjugate under nonreducing conditions. **d.** The downstream processing workflow of fermentation broth. **e.** The purified mn4-1BBL were separated by SDS-PAGE under nonreducing conditions and identified by Coomassie blue staining. M: prestained protein marker.


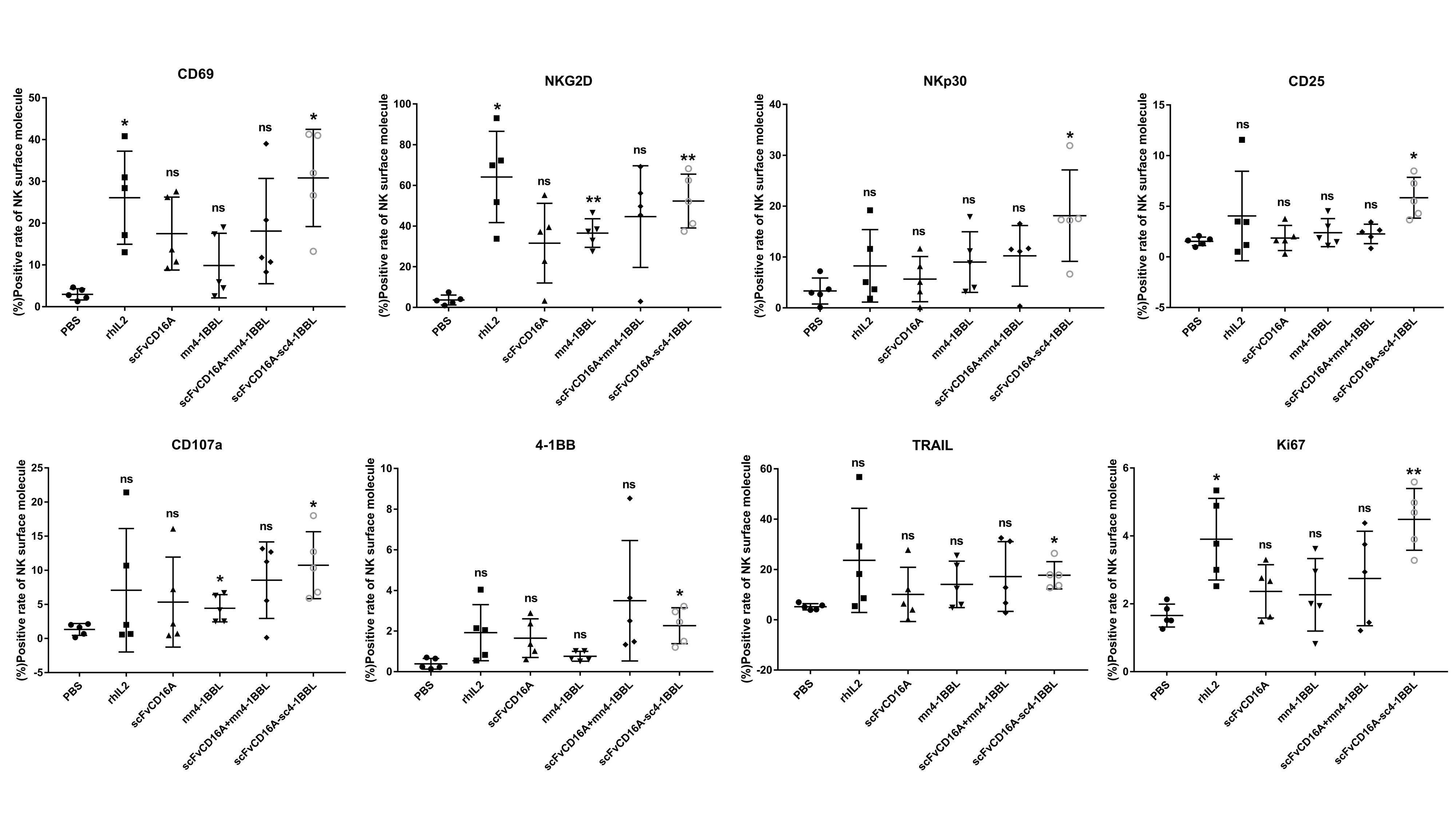
**Supplementary Fig 5:** **The expression level of NK surface molecules after stimulated by scFvCD16A-sc4-1BBL, corresponding to Fig. 4.
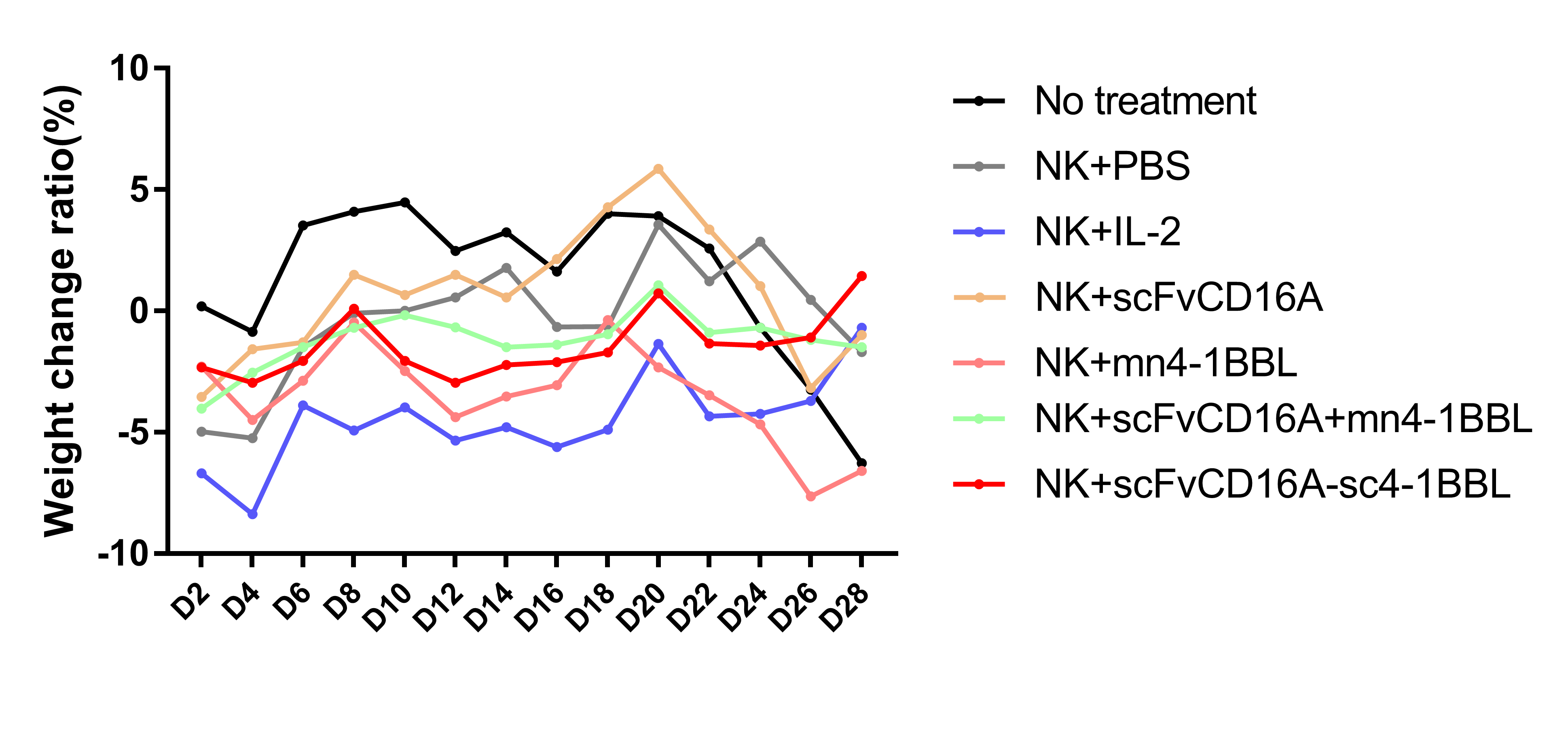
**

**Supplementary Fig 6:** **Monitoring curve of the weight change ratio in mice during the in vivo activity assay, corresponding to Fig. 6.**

.

**Supplementary Table 1** **The optimized parameters of culture conditions batch fermentation.**

| **Batch number** | **Feed rate(ml/h)** | **Temperature(℃）** | **pH** | **Dissolved oxygen(DO)** | **Agitator speed(rpm)** |
| --- | --- | --- | --- | --- | --- |
| ① | 90 | 25 | 7 | 25% | 1000 |
| ② | 70 | 25 | 7 | 25% | 800 |
| ③ | 80 | 25 | 7 | 25% | 800 |
| ④ | 80 | 25 | 6 | 25% | 800 |
| ⑤ | 80 | 25 | 6 | 25% | 1000 |
| ⑥ | 70 | 25 | 6 | 25% | 1000 |
